# *Tenacibaculum finnmarkense* sp. nov., a fish pathogenic bacterium of the family *Flavobacteriaceae* isolated from Atlantic salmon

**DOI:** 10.1007/s10482-015-0630-0

**Published:** 2015-12-11

**Authors:** Sverre Bang Småge, Øyvind Jakobsen Brevik, Henrik Duesund, Karl Fredrik Ottem, Kuninori Watanabe, Are Nylund

**Affiliations:** Fish Disease Research Group, Department of Biology, University of Bergen, Thormøhlensgt 55, Bergen, 5020 Norway; Cermaq Group AS, Dronning Eufemias Gate 16, Oslo, 0191 Norway; Cermaq Norway, Gjerbakknes, Nordfold, 8286 Norway

**Keywords:** Norway, Polyphasic taxonomy, Salmon farming, Skin lesions, Ulcerative disease, Winter ulcers

## Abstract

A novel Gram-stain negative, aerobic, non-flagellated, rod-shaped gliding bacterial strain, designated HFJ^T^, was isolated from a skin lesion of a diseased Atlantic salmon (*Salmo salar* L.) in Finnmark, Norway. Colonies were observed to be yellow pigmented with entire and/or undulating margins and did not adhere to the agar. The 16S rRNA gene sequence showed that the strain belongs to the genus *Tenacibaculum* (family *Flavobacteriaceae*, phylum *‘Bacteroidetes’*). Strain HFJ^T^ exhibits high 16S rRNA gene sequence similarity values to *Tenacibaculum dicentrarchi* NCIMB 14598^T^ (97.2 %). The strain was found to grow at 2–20 °C and only in the presence of sea salts. The respiratory quinone was identified as menaquinone 6 and the major fatty acids were identified as summed feature 3 (comprising C_16:1_ ω7c and/or iso-C_15:0_ 2-OH), iso-C_15:0_, anteiso-C_15:0,_ iso-C_15:1_ and iso-C_15:0_ 3-OH. The DNA G+C content was determined to be 34.1 mol%. DNA–DNA hybridization and comparative phenotypic and genetic tests were performed with the phylogenetically closely related type strains, *T. dicentrarchi* NCIMB 14598^T^ and *Tenacibaculumovolyticum* NCIMB 13127^T^. These data, as well as phylogenetic analyses, suggest that strain HFJ^T^ should be classified as a representative of a novel species in the genus *Tenacibaculum*, for which the name *Tenacibaculum finnmarkense* sp. nov. is proposed; the type strain is HFJ ^T^ = (DSM 28541^T^ = NCIMB 42386^T^).

## Introduction

During an outbreak of an ulcerative disease in Atlantic salmon at a seawater site in Finnmark, Norway, long rod shaped bacteria were found to predominate in the skin lesions. One strain of these isolates designated HFJ^T^ is described in the present study. The 16S rRNA gene sequence showed that it belongs to the genus *Tenacibaculum* (family *Flavobacteriaceae*, phylum *‘Bacteroidetes’*) described by Suzuki et al. (2001). To date the genus comprises 21 species derived from a variety of marine environments and marine organisms (Kim et al. 2013; LPSN 2015). Several of the type strains in genus *Tenacibaculum* have been reported as pathogenic for fish or associated with disease in cultured marine fish (Wakabayashi et al. 1986; Hansen et al. 1992; Piñeiro-Vidal et al. 2008a, b; López et al. 2010; Piñeiro-Vidal et al. 2012). *Tenacibaculummaritimum*, the causative agent of marine tenacibaculosis, is the best known and most extensively studied fish pathogenic bacterium in the genus (Wakabayashi et al. 1986; Suzuki et al. 2001). The disease has been reported from Europe, Japan, North America and Australia and affects both wild and cultured fish, including Rainbow trout and Atlantic salmon (Toranzo et al. 2005; Avendaño-Herrera et al. 2006; Bruno et al. 2013). *T. maritimum* has never been isolated in cases of ulcerative disease in Norway (Olsen et al. 2011).

There has been a growing attention regarding the potential role of Norwegian *Tenacibaculum* spp. in causing ulcerative disease in sea-reared Atlantic salmon, as they are commonly identified from skin lesion in mixed cultures with the bacterium *Moritella viscosa* or as the apparent sole agent (Olsen et al. 2011; Bornø and Lie 2015). The aim of the present study was to determine the taxonomic position of the fish pathogenic *Tenacibaculum* strain HFJ^T^ using genetic, phenotypic and chemotaxonomic characterisations, a detailed phylogenetic investigation based on 16S rRNA gene sequences and concatenated housekeeping (HK) gene sequences, and DNA–DNA hybridization (DDH).

## Materials and methods

A total of 11 isolates from genus *Tenacibaculum* were included in the present study (Table 1). Strain HFJ^T^ was isolated in spring 2013 from a skin lesion of a diseased Atlantic salmon at a seawater site in Finnmark, Norway. *Tenacibaculum* sp. strains Tsp. 2–7 were collected from skin or gill of Atlantic salmon and cod in Norway. The type strains *Tenacibaculum dicentrarchi* NCIMB 14598^T^, *Tenacibaculum ovolyticum* NCIMB 13127^T^, *Tenacibaculumsoleae* NCIMB 14368^T^ and *T. maritimum* NCIMB 2154^T^ were obtained from *TheNational Collection of Industrial*, *Marine and Food Bacteria* (NCIMB). Subcultivation was performed on Marine agar (MA) (Difco 2216) plates at 16 °C for 48 h. The strains were preserved in CryoTube™ vials (Thermo scientific) at −80 °C.

**Table 1:** List of *Tenacibaculum* strains included in the present study

Bacterial species	Strain	Origin	Host	Tissue	Year
*Tenacibaculum* sp.	HFJ^T^	Norway	Atlantic salmon	Skin	2013
*Tenacibaculum* sp.	Tsp.2	Norway	Atlantic salmon	Skin	2013
*Tenacibaculum* sp.	Tsp.3	Norway	Atlantic salmon	Gill	2014
*Tenacibaculum* sp.	Tsp.4	Norway	Atlantic salmon	Skin	2013
*Tenacibaculum* sp.	Tsp.5	Norway	Atlantic salmon	Skin	2014
*Tenacibaculum* sp.	Tsp.6	Norway	Atlantic salmon	Skin	2009
*Tenacibaculum* sp.	Tsp.7	Norway	Farmed Atlantic cod	Skin	2009
*Tenacibaculum maritimum*	NCIMB 2154^T^	Japan	Red sea bream fingerling	Kidney	1977
*Tenacibaculum soleae*	NCIMB 14368^T^	Spain	Senegalese sole	Unknown	2007
*Tenacibaculum ovolyticum*	NCIMB 13127^T^	Norway	Atlantic halibut eggs	Eggs	1989
*Tenacibaculum dicentrarchi*	NCIMB 14598^T^	Spain	European sea bass	Skin	2009

The *Tenacibaculum* sp. strains were collected from Norwegian field cases, whereas the type strains were obtained from NCIMB

Draft genome sequencing of strain HFJ^T^ was carried out by BaseClear B.V (Leiden, The Netherlands.) using Illumina next generation sequencing on a HiSeq 2500™ platform. Extraction of the required concentration (>100 ng/µl) of genomic DNA was performed using an E.Z.N.A. tissue DNA kit™ (Omega Bio-Tek) following the cultured cells protocol. The draft genome sequence obtained for strain HFJ^T^ was used for the PCR primer design using primer-BLAST (Ye et al. 2012) and to verify obtained sequences for strain HFJ^T^.

Genomic DNA from all *Tenacibaculum* sp. strains listed in Table 1 was extracted using an E.Z.N.A. tissue DNA kit™ (Omega Bio-Tek) following the cultured cells protocol. PCR was performed using the 16S rRNA primers 27F and 1518R (Giovannoni et al. 1996) and specific primers for five HK genes (*atpD*, *fusA,pgk, rpoB*, and *tuf*) (Table 2). Amplification was based on a standard reaction mixture containing 2.5 µl Extra buffer, 1.25 mM deoxyribonucleotide triphosphates, 0.75 units (0.15 µL) *Taq* DNA polymerase (BioLabs, New England), 5 µM (1 µL) of forward and reverse primers; DNase-RNase free water was added to a final volume of 25 µL (16.85 µL H_2_O). Amplification was performed in a GeneAmp PCR system 2700 (Applied Biosystems) at 95 °C for 5 min; 35 cycles of 95 °C for 30 s, 58 °C for 30 s, 72 °C for 60-100 s, followed by 72 °C for 10 min. The PCR product was confirmed by gel electrophoresis and enzymatically purified using ExoStar 1-Step ^®^ (GE Healthcare Bio-Sciences Corp) in an Artik Thermal Cycler (Thermo Scientific) at 37 °C for 15 min and at 80 °C for 15 min. The sequencing reaction was performed using a BigDye^®^ version 3.1 reaction in an Arktik Thermal Cycler, at 96 °C for 5 min; 30 cycles of 96 °C for 10 s, 58 °C for 5 s and 60 °C for 4 min. The reaction was composed of a mixture of 5.5 µL deionized water, 1 µL BigDye^®^ Terminator 3.1 version sequencing buffer, 1 µL BigDye Terminator 3.1 version Ready Reaction Premix (2.5X) (Invitrogen), 3.2 pmol (1µL) forward and reverse primers and 1.5 µL purified PCR product. Sequencing was carried out by the Sequencing Facility at Høyteknologisenteret i Bergen (http://www.uib.no/seqlab). Samples were cleaned with Agencourt CleannSeq (Beckman Coulter, Inc.) before being sequenced in a 96-capillary 3730xl DNA Analyzer (Applied Biosystems). The software Vector NTI^®^ v.9.0 (Invitrogen) was used to assemble and align the obtained sequences.

**Table 2:** List of PCR primers used in present study

Target gene	Name	Sequence (5′–3′)	Source
16S rRNA	B27F	AGAGTTTGATCMTGGCTCAG	Giovannoni et al. (1996)
16S rRNA	A1518R	AAGGAGGTGATCCANCCRCA	Giovannoni et al. (1996)
*tuf*	Tb_tuf F1	ACCTCCTTCACGGATAGC	Present study
*tuf*	Tb_tuf R1	TTACGATCGTTCGAAGCCCC	Present study
*rpoB*	Tb_rpoB F1	ATYTCTCCAAAACGCTGACC	Present study
*rpoB*	Tb_rpoB R1	AAAACGAATCAAGGWACGAAYA	Present study
*rpoB*	Tb_rpoB F2	ACCCTTTCCAAGGCATAAAGG	Present study
*rpoB*	Tb_rpoB R2	GAGCCATYGGTTTTGAAAGAGA	Present study
*rpoB*	Tb_rpoB F3	CTCTTGCTGTCTCCTCATCTG	Present study
*rpoB*	Tb_rpoB R3	ATCCACCAAGATATAGCATCCA	Present study
*pgk*	Tb_pgk F1	GCTCCWCCACCWGTAGAAAC	Present study
*pgk*	Tb_pgk R1	TYCGTGTAGATTTTAATGTGCCT	Present study
*atpD*	Tb_atpD F1	TGGYCCAGTWATCGATGTTGA	Present study
*atpD*	Tb_atpD R1	AATACGYTCTTGCATTGCTC	Present study
*fusA*	Tb_fusA F1	ATGGTAACTCACCCATTCCAGA	Present study
*fusA*	Tb_fusA R1	TGGCATGATGCAACACAAGG	Present study

Three alignments were constructed for phylogenetic analysis. The first, 16S rRNA gene sequence alignment, consisted of 1341 positions and included sequences of HFJ^T^ and the 21 published type strains in the genus *Tenacibaculum*. In this alignment, all sequences were obtained from GenBank with the exception of strain HFJ^T^, *T. dicentrarchi* NCIMB 14598^T^, *T. ovolyticum* NCIMB 13127^T^, *T. soleae* NCIMB 14368^T^ and *T. maritimum* NCIMB 2154^T^. The second 16S rRNA gene sequence alignment of 1349 positions contained sequences from all strains listed in Table 1. A third alignment, of 6750 positions, was constructed using concatenated sequences of the five HK genes of the strains listed in Table 1; *atpD* at position 1-807, *fusA* at position 808–1575, *pgk* at position 1576–2511, *rpoB* at position 2512–5778 and *tuf* at position 5779–6750. All sequences obtained in the present study are available in GenBank with accession numbers presented in Table 3. Alignments were constructed in AlignX in the Vector NTI^®^ v.9.0 (Invitrogen) software package before sequences were adjusted to equal length and correct reading frames in GeneDoc (Nicholas et al. 1997). Concatenation of the HK alignments was performed using KAKUSAN4 (Tanabe 2011). The best fitted evolutionary model for each alignment was calculated using Mega 6 (Tamura et al. 2013). For the Bayesian analysis of the concatenated HK alignment, KAKUSAN4 was used for calculation of substitution rate and the best fit model for the individual loci and codon positions and exported into a Mr. Bayes-block. A phylogenetic analysis of all three alignments were conducted using the Maximum Likelihood (ML) method with the best fitted evolutionary model, 1000 bootstrap replications and default settings in Mega 6. The BEAST package v1.8 (Drummond and Rambaut 2007) was used for Bayesian analysis of the two 16S rRNA gene datasets using the best fitted model, relaxed lognormal molecular clock and a mcmc of 100 000 000 generations. The Bayesian phylogenetic analysis of the HK gene dataset was conducted in Mr.Bayes V.3.2.2 (Ronquist et al. 2012) using the data block with the proportional codon proportional, model from KAKUSAN4 and a mcmc of 67 000 000 generations. *Kordia algicida* (GenBank accession nr: AB681152) was used as outgroup in the 16S rRNA phylogenetic analysis that included all the type strains, while *T. maritimum* NCIMB 2154^T^ was used as outgroup in the other phylogenetic analysis. The phylograms for the ML analysis were constructed in Mega 6. The Effective sample size values (ESS) in the Bayesian analysis were inspected using Tracer ver. 1.6 (Rambaut et al. 2014). All ESS values were within the recommended range (above 200) for all parameters. A maximum clade credibility tree was obtained using a 10 % burn-In in Tree-Annotator and viewed using FigTree (Drummond et al. 2012). For 16S rRNA gene sequence similarity analysis, Percent Nucleotide Identity (PNI) was calculated using the distance matrix option in BioEdit (Hall 2011). In the Average Nucleotide Identity (ANI) calculations, the sequences from the concatenated HK gene alignment for all strains listed in Table 1 were uploaded and analysed using the Average Nucleotide Identify option in EzGenome (Kim et al. 2012).

**Table 3:** List of GenBank accession numbers of sequences obtained in the present study

Bacterial species/strain	16S rRNA	*atpD*	*fusA*	*pgk*	*rpoB*	*tuf*
*T. finnmarkense* sp.nov HFJ^T^	KT270385	KT270377	KT270369	KT270424	KT270410	KT270399
*T. dicentrarchi* NCIMB 14598^T^	KT270381	KT270375	KT270364	KT270421	KT270408	KT270402
*T. maritimum* NCIMB 2154^T^	KT270382	KT270378	KT270366	KT270416	KT270411	KT270393
*T. ovolyticum* NCIMB 13127^T^	KT270383	KT270379	KT270367	KT270423	KT270412	KT270395
*T. soleae* NCIMB 14368^T^	KT270384	KT270380	KT270368	KT270417	KT270413	KT270394
*Tenacibaculum* sp. Tsp.2	KT270386	KT270376	KT270365	KT270420	KT270409	KT270400
*Tenacibaculum* sp. Tsp.3	KT270387	KT270373	KT270362	KT270422	KT270406	KT270397
*Tenacibaculum* sp. Tsp.4	KT270388	KT270370	KT270359	KT270414	KT270403	KT270392
*Tenacibaculum* sp. Tsp.5	KT270389	KT270371	KT270360	KT270418	KT270404	KT270398
*Tenacibaculum* sp. Tsp.6	KT270390	KT270372	KT270361	KT270415	KT270405	KT270396
*Tenacibaculum* sp. Tsp.7	KT270391	KT270374	KT270363	KT270419	KT270407	KT270401

Morphological, physiological and biochemical tests were performed as described by Bernardet et al. (2002) for strain HFJ^T^ and the phylogenetically closely related type strains *T. dicentrarchi* NCIMB 14598^T^ and *T. ovolyticum* NCIMB 13127^T^ as reference strains. All tests were performed on cultures incubated at 16 °C for 48 h unless otherwise stated. Colony shape, margin, elevation, size, texture, appearance, pigmentation and optical property were examined as described by Smibert and Krieg (1994). The ability to stick to agar and viscosity of the colonies was also investigated. Cell morphology was investigated using scanning electron microscopy (SEM), transmission electron microscopy (TEM) and light microscopy. Gliding motility was determined by phase contrast microscopic examination of a Marine broth (MB) (Difco 2216) culture by the hanging drop technique as recommended by Bernardet et al. (2002). Presence of flexirubin type pigments was determined by the bathochromic shift test using a 20 % (w/v) KOH solution (Fautz and Reichenbach 1980). Congo red absorption was tested as described by Bernardet et al. (2002). The Gram reaction were performed with a Fluka 77730 Gram Staining Kit (Fluka^®^ analytical) following the manufacturer`s protocol and the non-staining KOH method (Buck 1982). The Voges-Proskauer reaction was performed as described by Piñeiro-Vidal et al. (2012). Oxidase activity and ability to split indole from tryptophan was tested using BBL™ DrySlide Oxidase and BBL™ DrySlide Indole (BD BBL™, U.S.A), following the manufacturer`s protocol. Catalase activity was examined using the slide (drop) method following the protocol by Reiner (2010). Growth under anaerobic conditions was tested on MA using the GasPak anaerobic system (BBL). Production of H_2_S was detected by taping a lead acetate impregnated paper strip (Sigma) to the inside of the lid of MA plates, using Parafilm to seal lid and plate. The plates were incubated at 16 °C for 6 days. Growth on blood agar was tested using blood agar containing 2 % NaCl (BAS) (Microbial laboratory, Haukeland University Hospital, Bergen). Degradation of starch (1 % w/v), casein (1 % w/v), and Tween 80 (1 % v/v) was investigated on MA. MB supplemented with gelatin (1 % w/v) was used to investigate degradation of gelatin. Utilisation of carbon sources was tested on basal agar medium [0.2 g NaNO_3_, 0.2 g NH_4_Cl, 0.05 g yeast extract, 15 g agar and 36 g red sea salt (Red Sea)] per liter distilled water containing 0.4 % carbon source [d(+)-sucrose, d(−)-ribose, d(+)galactose, d-glucose, l-proline, l-glutamate, l-tyrosine] as described by (Suzuki et al. 2001). Absence of growth after one month of incubation was recorded as negative. Other enzymatic reactions were evaluated in the API ZYM system (bioMerieux) following the manufacturer’s instructions, except that sterile seawater was used as suspension medium. Growth at pH 4–10 (at unit intervals) was assessed in MB; pH was adjusted using 1 M NaOH and 1 M HCl. The temperature range for growth was determined on MA plates incubated at 2, 4, 8, 16, 20, 25, 30 and 37 °C for 7 days. Salinity requirement was determined with saltless MA [per liter distilled water: 5.0 g peptone, 1 g yeast extract and 0.1 g ferric citrate] containing 10, 20, 30, 50, 70 and 100 % strength seawater (100 % seawater = 38.2 g/L red sea salt) or 0.8, 1.0, 3.0, 5.0, 7.0 and 10.0 % (w/v) NaCl (Sigma). Sensitivity to antimicrobials was evaluated by the disc diffusion method following the procedures of *The Clinical and Laboratory Standards Institute* (CLSI 2005), except that the plates were incubated at 16 °C for 10 days on MA plates due to reduced growth for some strains on the recommended Flexibacter Maritimus Medium (FMM). The tests were performed using commercial discs (Neo-sensitabs™ and Sensi-disc™) containing kanamycin (500 µg), streptomycin (10 µg), gentamicin (30 µg), trimethoprim + sulfamethoxazole (125 + 2375 µg), ceftazidime (30 µg), ciprofloxacin (5 µg), pipemidic acid (30 µg), cefuroxime (30 µg), penicillin G (1U), ampicillin (2 µg), tetracycline (30 µg), erythromycin (15 µg), florfenicol (30 µg), oxolinic acid (10 µg) and oxytetracycline (30 µg). Several of the tests described above were also performed for the other strains included in the present study, except Tsp.7. Strain Tsp.7 was uncultivable after prolonged cryo-storage and was therefore not included in the phenotypic tests.

The following chemotaxonomic and genetic analyses were carried out by the Identification Service of the DSMZ (Braunschweig, Germany): DNA G+C content, DDH, menaquinone and fatty acid methyl ester analysis. All strains were grown in MB at 16 °C for 48 h, except for the DDH test. For DDH, cells were grown in MB at 16 °C for 72 h and the obtained bacterial biomass washed twice in 1× Phosphate Buffered Saline. The cells were disrupted using a Constant Systems TS 0.75 KW (IUL Instruments, Germany) and the DNA in the crude lysate purified by chromatography on hydroxyapatite as described by Cashion et al. (1977). DDH was carried out as described by Ley et al. (1970) under consideration of the modifications described by Huss et al. (1983) using a model Cary 100 Bio UV/VIS-spectrophotometer equipped with a Peltier-thermostatted 6 × 6 multicell changer and a temperature controller with in situ temperature probe (Varian). Extraction of fatty acid methyl esters, washing of extracts and GC analysis were performed by using the Sherlock MIS (MIDI Inc, Newark, USA) system using the MIDI Sherlock version 6.1 and TSBA40 database.

## Results and discussion

A polyphasic approach that integrates phenotypic data with genetic and phylogenetic data was performed in the current study. This approach is recommended by several authors for bacterial taxonomic investigations (Bernardet et al. 2002; Tindall et al. 2010). As it has been regarded as best practice to include more than one representative strain when describing a novel taxon, several *Tenacibaculum* sp. strains (Tsp.2–7) obtained from Norwegian mariculture were included in the present study. Strains HFJ^T^ and Tsp.2 have been shown to be pathogenic to Atlantic salmon reproducing the clinical signs in a challenge study in 2013 (Vold 2014). The bacteria were re-isolated and their identity confirmed by sequencing of the 16S rRNA gene, thus fulfilling Koch’s postulates.

**Fig. 1:**
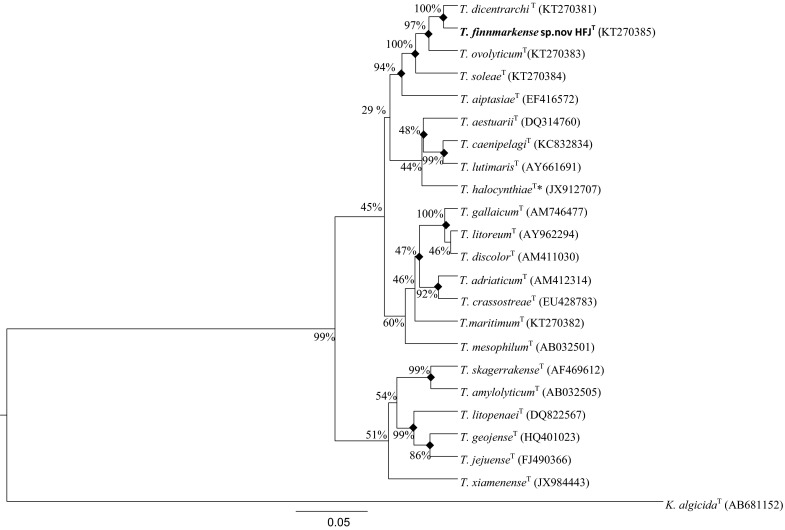
The relationship of the novel species *T. finnmarkense* sp. nov HFJ^T^ and the 21 type strains in genus *Tenacibaculum* (^*^ = quotation marks denote names that have not been validly published) based on the 16S rRNA gene sequences, using *Kordia algicida* ^T^ as outgroup. The phylogenetic analysis was inferred using the Bayesian method with the best fitted evolutionary model (GTR + G + I). The posterior probability is presented next to each node in percentage. There were a total of 1341 positions in the dataset. Evolutionary analyses were conducted using BEAST package v1.8. Shared nodes identified in corresponding ML analysis are marked with filled squares. Accesion numbers are in parentheses. *Scale bar* 0.05 substiutions per site

**Fig. 2:**
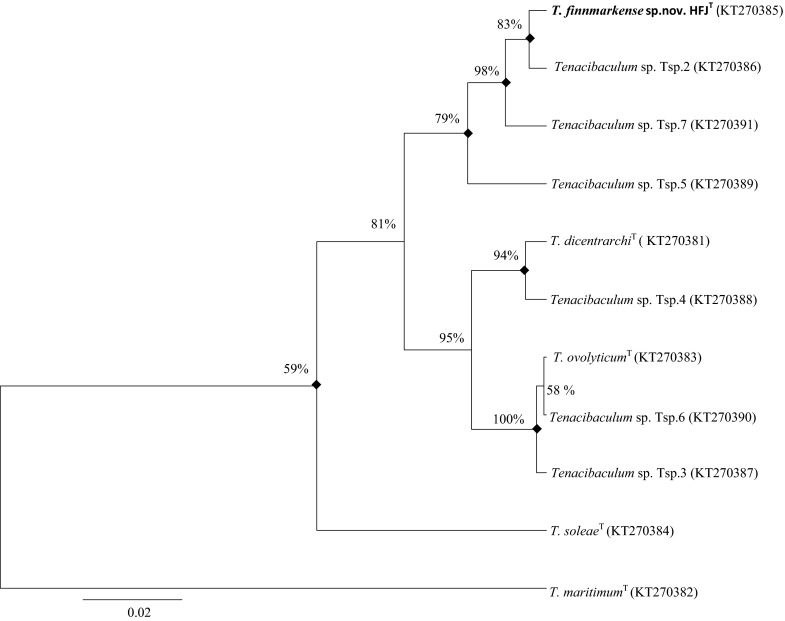
The relationship of the novel species *T. finnmarkense* sp.nov HFJ^T^, *Tenacibaculum* sp. strains Tsp. 2–7 and the three closest related type strains based on 16S rRNA gene sequences, using *T. maritimum* NCIMB 2154^T^ as outgroup. The phylogenetic analysis was inferred using the Bayesian method with the best fitted evolutionary model (HKY + G + I). The posterior probability is presented next to each node in percentage. There were a total of 1349 positions in the dataset. Evolutionary analyses were conducted using BEAST package v1.8. Shared nodes identified in corresponding ML analysis are marked with filled squares. Accesion numbers are in parentheses. *Scale bar* 0.02 substitutions per site

**Fig. 3:**
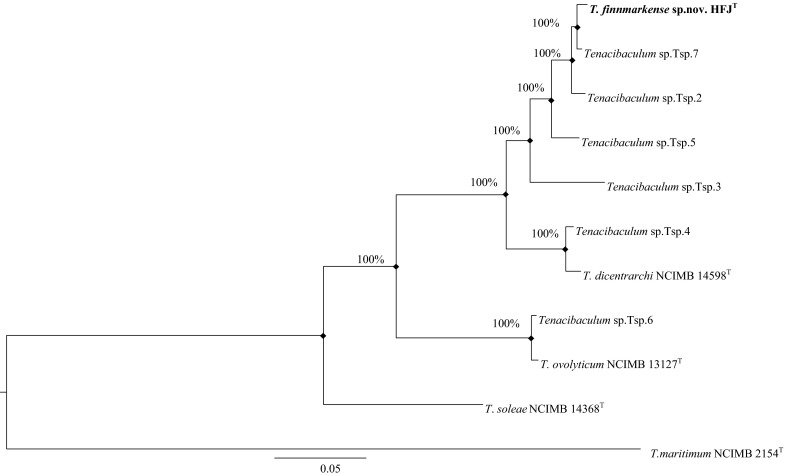
The relationship of the novel species *T. finnmarkense* sp.nov HFJ^T^, *Tenacibaculum* sp. strains Tsp. 2–7 and the three closest related type strains based on a concatenated sequences of five HK genes (*atpD* at position 1–807, *fusA* at position 808–1575, *pgk* at position 1576–2511, *rpoB* at position 2512–5778 and *tuf* at position 5779–6750), using *T. maritimum* ^T^ as outgroup. The accession numbers for the HK genes used in the concatenated dataset are presented in Table 3. The phylogenetic analysis was inferred using the Bayesian method with the best fitted evolutionary model. The posterior probability is presented next to each node in percentage. There were a total of 6750 positions in the dataset. Evolutionary analyses were conducted using KAKUSAN4 and Mr.Bayes. Shared nodes identified in corresponding ML analysis are marked with filled squares. *Scale bar* 0.05 substitutions per site

The phylogenetic analysis based on the 16S rRNA gene sequences and the concatenated HK gene sequences (Fig. 1, 2, 3) showed that strains HFJ^T^, Tsp.2, Tsp.5 and Tsp.7 belong to a distinct clade separate from the closely related type strains in the genus *Tenacibaculum*. Moreover, the analysis showed that strain Tsp.4 forms a clade with *T*. *dicentrarchi* NCIMB 14598^T^ and Tsp.6 forms a clade with *T. ovolyticum* NCIMB 13127^T^, while the phylogenetic placement of strain Tsp.3 is uncertain. These clades were evident in all phylogenetic trees using both the Bayesian and ML method. All phylogenetic trees showed that strain HFJ^T^ is closely related to *T. dicentrarchi* NCIMB 14598^T^, both belonging to distinct clades.

As strain HFJ^T^ showed more than 97 % 16S rRNA gene sequence similarity (PNI) to *T*. *dicentrarchi* NCIMB 14598^T^ (Table 4), DDH was performed as recommended (Stackebrandt and Goebel 1994; Tindall et al. 2010). The DDH tests revealed that the DNA relatedness of strain HFJ^T^ was 54.8 (52.0) % to *T. dicentrarchi* NCIMB 14598^T^ and 36.6 (39.7) % to *T. ovolyticum* NCIMB 13127^T^. Results from repeated tests are shown in parentheses. When considering the threshold value of 70 % DNA–DNA similarity for delineation of bacterial species proposed by the *ad hoc* committee (Wayne et al. 1987), strain HFJ^T^ does not belong to the species *T. dicentrarchi* NCIMB 14598^T^ or *T. ovolyticum* NCIMB 13127^T^. It is generally accepted that an ANI value of 95–96 % corresponds to a DDH threshold value of 70 % and can be used as a boundary for species delineation (Goris et al. 2007; Richter and Rosselló-Móra 2009). Furthermore, a study by Kim et al. (2014) revealed that a PNI of 98.65 % corresponded to an ANI value of 95–96 %. The calculated ANI and PNI values between strain HFJ^T^ and *T. dicentrarchi* NCIMB 14598^T^ were 94.6 and 97.2 % respectively. By applying both the ANI and PNI threshold on all 11 strains included in this study (Table 4) we found that strains HFJ^T^, Tsp.2, Tsp.5 and Tsp.7 belong to the same species; Tsp.4 belongs to the species *T. dicentrarchi*, while Tsp.6 belongs to the species *T. ovolyticum*. These findings correspond to results from the phylogenetic analysis and underpin that strain HFJ^T^ represents a novel species in genus *Tenacibaculum.*

**Table 4:** Results from the PNI and ANI analysis performed for all strains listed in Table 1

	Tsp.2	Tsp.3	Tsp.4	Tsp.5	Tsp.6	Tsp.7	Strain HFJ^T^	*T.dicentrarchi* ^T^	*T.ovolyticum* ^T^	*T.soleae* ^T^	*T.maritimum* ^T^
Strain HFJ^T^	99.3 (98.7)	95.7 (95.4)	97.3 (94.8)	98.6 (97.4)	96.0 (91.2)	98.8 (99.3)		97.2 (94.6)	96.0 (91.1)	96.4 (89.5)	93.6 (83.7)
*T. dicentrarchi* ^T^	97.1 (94.6)	96.8 (94.0)	99.6 (99.0)	98.2 (95.2)	97.0 (90.7)	97.0 (94.6)	97.2 (94.6)		97.0 (90.6)	96.5 (89.3)	93.1 (84.4)
*T. ovolyticum* ^T^	96.6 (91.1)	99.7 (91.0)	97.0 (90.6)	96.3 (90.9)	100 (99.4)	96.8 (91.0)	96.0 (91.1)	97.0 (90.6)		96.7 (89.8)	93.2 (85.2)
*T. soleae* ^T^	96.6 (89.6)	96.5 (89.4)	96.3 (89.3)	96.9 (89.5)	96.7 (89.8)	97.1 (89.5)	96.4 (89.5)	96.5 (89.3)	96.7 (89.8)		94.0 (84.3)
*T. maritimum* ^T^	93.7 (83.9)	92.9 (83.9)	93.1 (84.2)	93.3 (84.0)	93.2 (85.3)	93.8 (84.0)	93.6 (83.7)	93.1 (84.4)	93.2 (85.2)	94.0 (84.3)	

The pairwise 16S rRNA sequence identities and ANI values are presented as percent (%) similarity. ANI values are shown in parentheses

Cells of strain HFJ^T^ were observed to be rod-shaped, 0.5 µm wide and 5–25 µm in length and Gram-stain negative. Considerably longer filamentous cells and spherical degenerative cells were frequently observed in older cultures. A rapid decrease in viability was found to occur with prolonged incubation (>96 h). Differential phenotypic characteristics between all strains listed in Table 1, except strain Tsp.7, are summarised in Table 5 and are included in the species description. The G+C content of strain HFJ^T^ was determined to be 34.1 mol% which is within the range reported for other type strains in the genus *Tenacibaculum* (29.8–35.2 mol%). The major fatty acids (>5 % of the total fatty acids) for strain HFJ^T^ were identified as summed feature 3 (comprising C_16:1_ ω7c and/or iso-C_15:0_ 2-OH), iso-C_15:0_, anteiso-C_15:0,_ Iso-C_15:1_ and iso-C_15:0_ 3-OH. Results from the fatty acid analysis for strain HFJ^T^ and *T. dicentrarchi* NCIMB 14598^T^ are listed in Table 6. The respiratory quinone was identified as menaquinone 6 (100 %) while flexirubin-type pigments were found to be absent. This is in accordance with the chemotaxonomic characteristics of the members of the genus *Tenacibaculum* (Suzuki et al. 2001). In the API ZYM system, alkaline phosphatase, esterase (C4), esterase lipase (C8), leucine arylamidase, valine arylamidase, cystein arylamidase, acid phosphatase and naphthol-AS-BI-phosphohydrolase were found to be present. Lipase (C14), trypsin, α-chymotrypsin and all enzymes related to the metabolism of carbohydrates were found to be absent. Strain HFT^T^ was found to be susceptible to trimethoprim-sulfamethoxazole, ceftazidime, ciprofloxacin, pipemidic acid, cefuroxime, penicillin G, ampicillin, tetracycline, erythromycin, florfenicol, oxytetracycline and oxolinic acid, but resistant to kanamycin, gentamicin and streptomycin.

**Table 5:** Differential characteristics of all strains listed in Table 1, except strain Tsp.7

Characteristic	Tsp.2	Tsp.3	Tsp.4	Tsp.5	Tsp.6	HFJ^T^	*T.dicentrarchi* ^T^	*T.ovolyticum* ^T^	*T.soleae* ^T^	*T.maritimum* ^T^
Cell size	3–30 µm	2–20 µm	2–40 µm	2–25 µm	2–15 µm	5–25 × 0.5 µm	2–40 µm	2–10 µm	2–25 µm	2–30 µm
Gram stain	−	−	−	−	−	−	−	−	−	−
Gliding motility	+	+	+	+	+	+	+	+	+	+
Colony morphology	Circular	Circular	Circular	Circular	Circular	Circular	Circular	Circular	Circular	Circular
Color	Pale yellow	Bright Yellow	Pale yellow	Yellow	Pale yellow	Yellow	Brownish yellow	Pale yellow	Yellow	Pale yellow
Growth temp °C	8–16^b^	8–16^b^	8–16^b^	8–16^b^	8–16^b^	2–20	2–25	8–25	8–16^b^	8–16^b^
Salinity range										
NaCl	Nt	Nt	Nt	Nt	Nt	Ng	Ng	Ng	Nt	Nt
Seawater %^a^	Nt	Nt	Nt	Nt	Nt	50–100	50–100	70–100	Nt	Nt
pH range	Nt	Nt	Nt	Nt	Nt	4–9	5–10	Nt	Nt	Nt
Catalase	w	w	w	w	w	w	w	w	w	+
H_2_S	Nt	Nt	Nt	Nt	Nt	−	+	+	Nt	Nt
Antimicrobial drugs										
Ceftazidime	s	r	s	s	r	s	s	r	r	s
Pipemidic acid	s	s	s	s	s	s	s	s	r	s
Penicillin G	s	r	s	s	r	s	s	r	r	s
Ampicillin	s	s	s	s	r	s	s	r	r	s
Growth on										
l-tyrosin	Nt	Nt	Nt	Nt	Nt	−	−	+	Nt	Nt
d-glucose	Nt	Nt	Nt	Nt	Nt	−	−	+	Nt	Nt
Blood agar (2 % NaCl)	−	+	−	−	−	−	+^c^	−	+^d^	+
Hydrolization of										
Tween 80	Nt	Nt	Nt	Nt	Nt	−	+	+	Nt	Nt
API-ZYM										
Esterase (C4)	+	+	+	+	+	+	+	−	+	−
Cystein arylamidase	+	+	+	+	+	+	+	−	−	−
Trypsin	−	+	−	−	+	−	−	+	−	−
N-acetyl-β-glucosaminidase	−	+	−	−	+	−	−	+	−	−
G+C (mol%)	Nt	Nt	Nt	Nt	Nt	34.1	31.3	30.3	Nt	Nt

All data is from this study, except the DNA G+C contents of the two reference strains taken from Piñeiro-Vidal et al. (2012) and Suzuki et al. (2001)

+ positive, − negative, *w* weakly positive, *Nt* not tested, *Ng* no growth, *r* resistant, *s* susceptible. All strains are oxidase positive and indole negative

^a^Percent calculated using a relation of 100 % seawater = 38.2 g red sea salt L^−1^

^b^Only tested at 8 and 16 °C

^c^Induces β-hemolysis or hemedigestion (CDC 2013) on blood agar containing 2 % NaCl

^d^Induces α-hemolysis on blood agar containing 2 % NaCl

**Table 6:** Cellular fatty acid composition (%) of strain HFJ^T^ and *T. dicentrarchi* NCIMB 14598^T^

Fatty acid	1	2
Straight chain		
C_14:0_	1.3	1.0
C_15:0_	1.8	3.8
Branched chain		
iso-C_13:0_	1.3	1.3
iso-C_14:0_	1.6	2.5
iso-C_15:0_	17.1	15.2
anteiso-C_15:1_	17.7	13.3
iso-C_15:1_	9.5	9.0
anteiso-C_15:1_	1.9	1.9
iso-C_16:0_	Tr	1.0
iso-C_16:1_	Tr	2.8
Unsaturated		
C_15:1_ω6c	3.3	3.1
C_16:1_ω5c	1.8	1.6
C_17:1_ω6c	Tr	2.0
Hydroxylated		
iso-C_15:0_ 3-OH	12.4	11.6
C_15:0_ 2-OH	1.0	1.3
C_15:0_ 3-OH	1.5	2.2
iso-C_16:0_ 3-OH	2.9	5.3
C_16:0_ 3-OH	4.0	3.8
iso-C_17:0_ 3-OH	2.4	2.7
Summed feature 3^a^	9.5	10.3

*Strains* *1* HFJ^T^, *2* *T. dicentrarchi* NCIMB 14598^T^

All data are from this study. Fatty acids amounting to <1 % of the total fatty acids in all strains are not shown. Tr, Trace (<1 %)

^a^Summed feature are groups of two or three fatty acids that cannot be separated by GLC using the MIDI system. Summed feature 3 comprises C_16:1_ ω7c and/or iso-C_15:0_ 2-OH

Results from the phenotypic and chemotaxonomic tests show that strain HFJ^T^ differs significantly from *T. dicentrarchi* NCIMB 14598^T^ and *T. ovolyticum* NCIMB 13127^T^ (Table 5). The fatty acid composition analysis (Table 6) shows that strain HFJ^T^ has a very similar profile compared to that of *T. dicentrarchi* NCIMB 14598^T^. Moreover, the G+C content of strain HFJ^T^ is higher than those reported for *T. dicentrarchi* NCIMB 14598^T^ and *T. ovolyticum* NCIMB 13127^T^. Strain HFJ^T^ and *T. ovolyticum* NCIMB 13127^T^ do not grow on BAS, in contrast to *T. dicentrarchi* NCIMB 14598^T^. *T. ovolyticum* NCIMB 13127^T^ is positive for the enzymes trypsin and N-acetyl-glucosaminidase, while strain HFJ^T^ and *T. dicentrarchi* NCIMB 14598^T^ are negative. *T. ovolyticum* NCIMB 13127^T^ was unique in being resistant to the antimicrobial drugs ceftazidime, penicillin G and ampicillin. The above mentioned differences further support strain HFJ^T^ as representative of a novel species in the genus *Tenacibaculum*. Cell length was the only characteristic shown to correspond to the three clades inferred in the phylogenetic analysis. Results showed a length of 2–40 µm for strain Tsp.4 and *T. dicentrarchi* NCIMB 14598^T^, 2–30 µm for strains HFJ^T^, Tsp.2, and Tsp.5, and 2–15 µm for strain Tsp.6 and *T. ovolyticum* NCIMB 13127^T^.

In conclusion, the differential genetic, phylogenetic, phenotypic and chemotaxonomic data presented shows that strain HFJ^T^ should be classified as a novel species in genus *Tenacibaculum,* for which the name *Tenacibaculum finnmarkense* sp.nov. is proposed. This novel species also includes strains Tsp.2 and Tsp.5.

### Description of *Tenacibaculum finnmarkense* sp. nov.

*Tenacibaculum finnmarkense* (finn.mark.en’se. N.L. neut.adj. *finnmarkense* of Finnmark, Norway, referring to the place of isolation).

Cells are strictly aerobic, Gram-stain negative, straight rods, 0.5 µm in diameter and 2–30 µm in length (filamentous cells >100 µm long may occur in older cultures) and motile by gliding. Degenerative spherical cells are observed in ageing cultures. Colonies on MA are circular, convex, pale yellow or yellow pigmented with translucent edges, have entire and/or undulating margins and a smooth texture with a shiny and sometimes nacreous appearance. The colonies are slightly viscous and do not stick to agar. Congo red absorption is negative. Growth occurs in media containing 50–100 % strength seawater but not in media supplemented with NaCl only. No growth occurs on BAS. Growth occurs at 2, 4, 8, 16 and 20 °C, but not at 25, 30 and 37 °C. Growth occurs at pH 4.0–9.0 (optimum pH 6–8). Catalase and cytochrome oxidase activities are present. Gelatin and casein are hydrolysed, but Tween 80 and starch are not. The Voges–Proskauer and flexirubin tests are negative. No anaerobic growth is observed. H_2_S and indole are not produced. l-Proline and l-glutamate are utilised but d(+)-sucrose, d(−)-ribose, d(+)-galactose, d(+)-glucose and l-tyrosine are not. The major fatty acids (>5 % of the total fatty acids) are summed feature 3 (comprising C_16:1_ ω7c and/or iso-C_15:0_ 2-OH), iso-C_15:0_, anteiso-C_15:0,_ Iso-C_15:1_ and iso-C_15:0_ 3-OH. The respiratory quinone is menaquinone 6. The DNA G+C content of the type strain is 34.1 mol%.

The type strain is HFJ^T^ (=DSM 28541^T^ = NCIMB 42386^T^), isolated from diseased Atlantic salmon (*Salmo salar* L.) in Norway. The GenBank accession number for the 16S rRNA gene sequence of strain HFJ^T^ is KT270385.
